# Hypoxic hepatitis as a complication of newly diagnosed type 1 diabetes in a teenager

**DOI:** 10.4322/acr.2021.372

**Published:** 2022-04-14

**Authors:** Kamil Buczkowski, Irena Ożóg-Zabolska, Jacek Gulczyński, Ewa Iżycka-Świeszewska

**Affiliations:** 1 Szpital im. Mikołaja Kopernika (COPERNICUS PL), Department of Pathomorphology, Gdansk, Poland; 2 Gdański Uniwersytet Medyczny (GUMed), Department of Pathology and Neuropathology, Gdansk, Poland; 3 Szpital im. Mikołaja Kopernika (COPERNICUS PL), Pediatric Intensive Care Unit, Gdansk, Poland

**Keywords:** Hepatitis, Gastroenterology, Pediatrics, Diabetes Mellitus, Autopsy

## Abstract

Hypoxic hepatitis is a rare complication of type 1 diabetes with unknown prevalence in Pediatrics. We present a case report of an 11-year-old boy admitted to the ER in the spring of 2020 (the beginning of the COVID19 pandemic in Poland) due to nausea, abdominal pain, and weight loss. A diagnosis of type 1 diabetes accompanied by severe ketoacidosis (pH 6.9, blood glucose 632mg/dl, ketone bodies in urine – 150mg/dl) was made. The hyperglycemia, ketoacidosis, and water-electrolyte disturbances were treated in the Pediatric Intensive Care Unit. On day 4, the boy developed fulminant septic shock with high aminotransferases (AST 9026 U/l, ALT 3559 U/l). CT scan revealed hepatic enlargement and steatosis. Acute viral hepatitis was suspected. The levels of anti-CMV IgM and IgG antibodies were slightly elevated. At autopsy, the liver was enlarged, with petechial bleedings on the surface. The liver parenchyma was congested, with signs of steatosis. Microscopically, there was extensive centrilobular necrosis, acute passive sinusoidal congestion, and steatosis of hepatocytes. There were no signs of CMV infection. Based on the entire clinicopathological picture, the patient was diagnosed with hypoxic hepatitis, complicated by septic shock and multiple organ failure.

## INTRODUCTION

The incidence of type 1 diabetes in Pediatrics steadily increases and currently affects approximately 15 per 100,000 patients in Europe and 20 per 100,000 patients in America.^1^^,^^2^ One of its first manifestations may be diabetic ketoacidosis, which occurs in approximately 33% of cases of newly diagnosed diabetes.^3^ Diabetic ketoacidosis can manifest as polyuria, polydipsia, hyperventilation, Kussmaul's breath, breath smell like acetone, nausea, vomiting, abdominal pain, and impaired consciousness.^4^^,^^5^ Untreated ketoacidosis can lead to acute pancreatitis, thromboembolic episodes, pulmonary edema, acute renal failure, hypovolemic shock, rhabdomyolysis, and cerebral edema, which is the leading cause of death in these patients.^6^^,^^7^ One of the very rare complications of severe diabetic ketoacidosis may be hypoxic hepatitis.^8^

## CASE REPORT

An 11-year-old boy was admitted to the Emergency Department in the spring of 2020 due to vomiting, severe abdominal pain, and fever over the last two days, with no signs of active infection. SARS-CoV-2 was ruled out. Medical history revealed polyuria and polydipsia for more than two weeks. The boy had lost 9 kg in the preceding month. On admission, he was severely ill; however, he was conscious but slightly confused. Laboratory tests showed a pH of 6.9 (RR: 7.35-7.45) blood glucose level of 632 mg/dl (RR: 70-99mg/dl). The urinalysis showed a glucose level of 1000mg/dl (RR: negative) and a ketone body level of 150 mg/dl (RR: negative).

The patient was diagnosed with type 1 diabetes mellitus and severe ketoacidosis and was referred to the Pediatric Intensive Care Unit.

**Figure 1:**
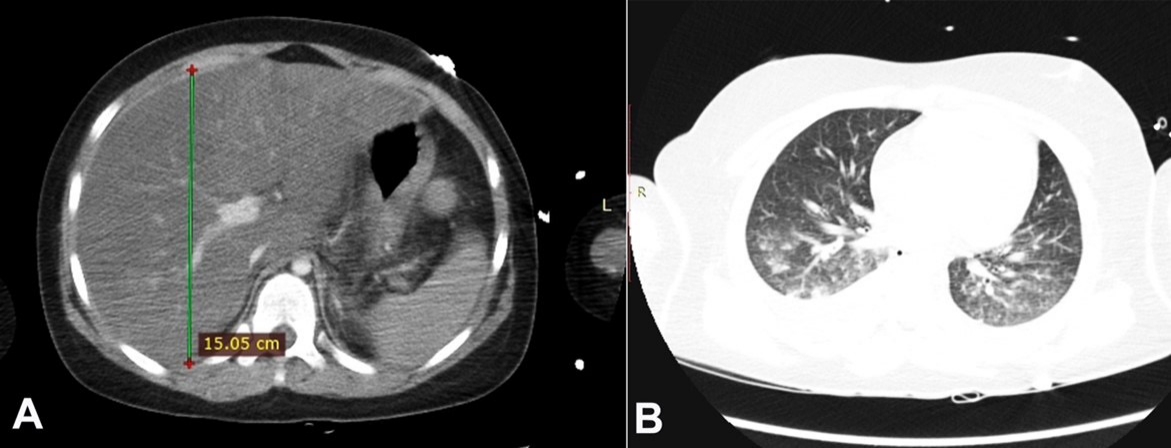
**A –** The abdominal CT scan shows unevenly distributed liver steatosis and hepatomegaly; **B –** CT scan of the lungs shows peribronchial parenchymal consolidation and a small amount of fluid in both pleural cavities.

Metabolic compensation and monitoring of the vital functions were promptly started. On admission, serum aminotransferases levels were: AST 10U/l (RR: 38U/I), ALT 9 U/l (RR:30U/I). During the first two days of hospitalization, serum glucose levels were lowered to 260mg/dl (RR: 70-99mg/dl), metabolic acidosis and sodium levels were equalized. Potassium levels remained slightly below the lower limit of normal. On the following day, consciousness deterioration and respiratory distress ensued. The patient was intubated and placed on mechanical ventilatory support. A brain CT scan was normal, and the blood cultures were negative. Due to the increase in inflammatory parameters, empiric broad-spectrum antibiotic therapy was initiated. On the fourth day (D4) of hospitalization, an increase of the abdominal girth and the elevation of aminotransferases were detected (ALT 3559 U/l (RR: 30U/I), AST 9026 U/l (RR: 38U/I)) (Table 1). A thoracic and abdominal contrast-enhanced CT scan was performed, which showed hepatomegaly (longitudinal dimension of the right lobe - 150.5mm (RR: 105mm), diffuse steatosis (liver attenuation was approximately 30 Hounsfield Units (HU) less than the spleen (RR: <10HU) (Figure 1A), and bilateral pulmonary parenchymal consolidations consistent with bronchopneumonia (Figure 1B). Liver vessels were patent. It also showed a blurred outline of the pancreas with a small amount of peripancreatic fluid, without typical features of acute pancreatitis.

**Table 1:** Laboratory tests performed in the Intensive Care Unit on day 1 and day 4 of hospitalization.

**Parameter**	**D1**	**D4**	**RR**	**Parameter**	**D1**	**D4**	**RR**
pH	6.9	6.85	7.35-7.45	AST	10	9026	<38 U/l
K+	4.2	5.6	3.5-5.1 mmol/l	Ammonia	-	820.14	<102 ug/dl
Na+	142	139	132-141 mmol/l	Creatinine	1.04	2.01	<0.79 mg/d
Glucose	632	129	70-99 mg/dl	WBC	27,740	9,710	4,500-13,500 /µl
Lactic acid	18	216	5-14 mg/dl	Neutrophils	20,360	6,250	1,800-7,700/µl
CRP	3.12	130.07	<5 mg/l	PLT	409,000	49,000	140,000-420,000/µl
Procalcitonin	-	16.8	<0.5 ng/ml	APTT	20.2	66.5	25.4-36.9 sec.
Amylase	27	138	<91 U/l	PT	13.2	38	9.4-12.5 sec.
ALT	9	3559	<30 U/l	INR	1.16	>6	0.9-1.3

ALT= alanine aminotransferase; AST= aspartate aminotransferase; APTT= activated partial thromboplastin time; CRP= C-reactive protein; D1= first day; D4= day four; PLT= platelet; PT= prothrombin time; RR= reference range; WBC= white blood cells;

Radiologists have suggested that the liver injury pattern could indicate an acute liver injury, such as acute viral hepatitis. The serum levels of anti-CMV, EBV, HSV1, HSV2, and parvovirus antibodies showed a slight increase of IgM and IgG CMV antibodies; the remaining were negative. On the same day, a sudden and unresponsive to catecholamines hypotension ensued. The laboratory tests showed platelets count of 49,000/µl (RR: 140,000-420,000/µl), creatinine 2.01mg/dl (RR: <0,79mg/dl), INR>6 (RR: 0.9-1.3), procalcitonin 16.8 ng/ml (RR: <0.5ng/ml) (Table 1). The patient developed septic shock and acute liver failure. The subsequent blood cultures were negative.

Despite the therapeutic efforts, the patient did not recover the normalization of the vitals with sustained lactic acidosis and died on the fifth day of hospitalization. The body was sent for autopsy with a request to rule out acute viral hepatitis or hepatic metabolic diseases.

## AUTOPSY PRESENTATION

Organ weight: brain: 1300g (RR:1100-1650g); heart: 150g (RR: 110-290g); kidneys (L / R): 100g/100g (RR:45-150g); liver: 1350g (RR:470-1800g); spleen: 110g (20-200g).

The brain was symmetrical and macroscopically normal. The trachea, main and segmental bronchi contained yellowish, purulent content. Lungs were edematous. The parenchyma was reddish, congested at the base, with bilateral greyish consolidations. The myocardium was pale. The kidneys were of normal shape and size, with a smooth surface. The cortex was pale and sharply demarcated from dark-red pyramids. The pancreas was grey-pink, with lobular structure, normal size, and consistency. The pancreatic duct was filled with thick mucus. The liver was enlarged, with a focally congested capsule. The parenchyma was yellowish-brown with a blurred, lobular structure, congested and friable on palpation.

**Figure 2:**
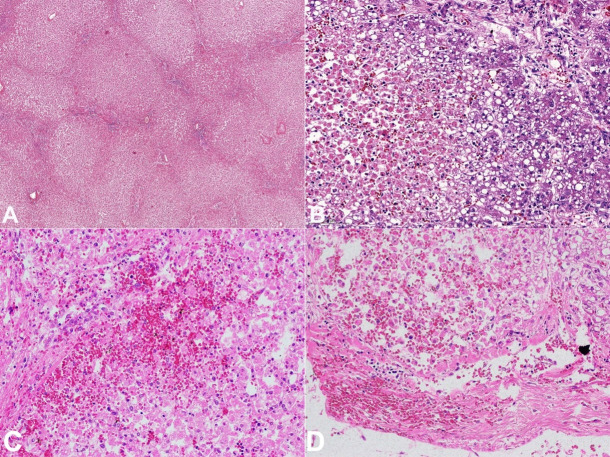
Photomicrographs of: **A –** Liver – the areas of centrilobular necrosis and passive and passive sinusoidal congestion with relatively preserved periportal hepatocytes (H&E, 4X); **B –** Early hepatocyte necrosis and passive sinusoidal congestion (left side) and preserved hepatocytes with features of steatosis (right side) (H&E, 20X); **C, D –** The areas of petechial bleedings in liver parenchyma and the subcapsular area (H&E, 40X).

**Figure 3:**
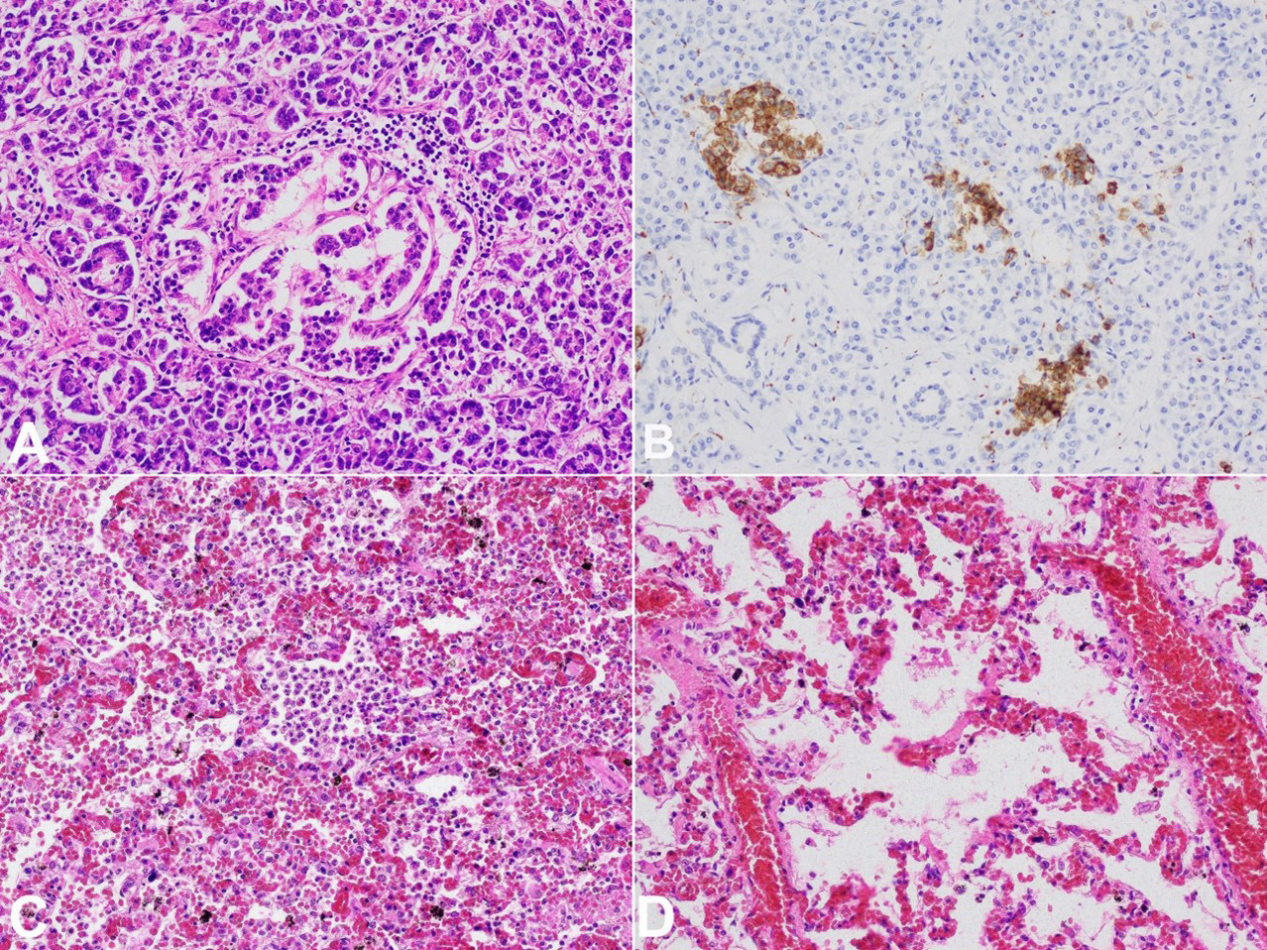
Photomicrographs of: **A –** Pancreas – pancreatic islet with disturbed architecture, surrounded by lymphocytic infiltrate (H&E, 40X); **B –** Pancreas - abnormal pancreatic islet structure highlighted by synaptophysin staining (20X); **C –** Lungs – area of early bronchopneumonia (H&E, 20X); **D –** Lungs - signs of passive congestion with hyaline membranes formation (H&E, 40X).

Intrahepatic bile ducts were not dilated, and the portal vein was patent. Multiple samples were taken during the autopsy. They were stained with H&E. The microscopic examination disclosed extensive early centrilobular necrosis associated with acute perivenular, and sinusoidal congestion. The tissue under the hepatic capsule had foci of petechial bleedings. Hepatocytes located in the periportal zone showed macrovesicular steatosis and intracellular cholestasis. Some of the hepatocytes also showed glycogen vacuoles in the nuclei. Sparse lymphocytic infiltrate was located in the portal triads. Multiple small thrombi were visible in small blood vessels. No features of cytomegalovirus infection or metabolic diseases were identified. The overall picture corresponded to hypoxic hepatitis (Figure 2A-D). Lungs showed features of ARDS/DAD (acute respiratory distress syndrome/diffuse alveolar damage) with deposits of hyaline membranes, areas of intra-alveolar hemorrhages, pulmonary edema, and early bronchopneumonia (Figure 3C, D).

A small hemorrhagic infarct was also identified. Pancreatic islets had disturbed architecture and focal, scanty lymphocytic infiltrate. Pancreatic ducts were filled with thick mucus deposits (Figure 3A, B). Kidneys presented early acute tubular necrosis. Moderate edema with signs of focal acute neuronal damage was the only finding within the brain.

## DISCUSSION

Clinically, hypoxic hepatitis is a reversible, acute liver injury characterized by a significant and rapid increase in serum aminotransferases.^9^ The serum bilirubin level is usually slightly elevated.^10^ It is most often associated with comorbidities predisposing to ischemia, venous congestion, arterial hypoxemia, and poorer hepatic oxygenation, such as congestive heart failure, ischemic heart disease, sepsis, shock, severe anemia, or acute respiratory failure.^11^^,^^12^ Criteria for the diagnosis of hypoxic hepatitis are based on: (1) the presence of risk factors predisposing to the development of hypoxic hepatitis; (2) a significant and transient increase in serum aminotransferases (usually more than 20-times the upper limit); (3) the exclusion of other possible causes of liver damage (e.g., drug-induced damage, viral hepatitis).^8^^,^^9^

Rapid increases in serum aminotransferases (ASAT, ALAT) and lactate dehydrogenase (LDH) levels occur shortly after the trigger and peak within 24-48h. In the next 24-72 hours, enzyme levels drop by half of their baseline and normalize within 2 weeks.^13^^,^^14^ The pathophysiological mechanism underlying hypoxic hepatitis is multifactorial and not fully understood. A cumulative effect of passive congestion, poor oxygen utilization, and reperfusion injury is usually suggested.^14^

Histopathologically, hypoxic hepatitis is characterized by centrilobular necrosis, sometimes extensive, accompanied by sinusoidal widening and acute passive congestion. Healthy hepatocytes are usually located around the portal triads and may show signs of steatosis.^13^ Differential diagnoses should include acute viral hepatitis, drug-induced liver injury, toxic liver injury, liver trauma, autoimmune hepatitis, hepatic infarction, Budd-Chiari syndrome, HELLP syndrome, acute obstruction of the biliary system, metabolic and genetic diseases.^9^^,^^14^^,^^15^ Imaging findings are uncharacteristic and usually show the consequences of acute congestion, such as dilatation of the hepatic veins. Although a liver biopsy and imaging techniques can contribute some information, they are not required to make a diagnosis.^9^

It is worth mentioning that one of the causes of hepatomegaly in children with poorly controlled type 1 diabetes may be Mauriac syndrome. In addition to hepatomegaly, it is characterized by diffuse nuclear glycogenization of hepatocytes, slight macrovesicular steatosis, growth retardation, cushingoid appearance, and hyperlipidemia. Aminotransferase levels are usually elevated, but most of the time is lower than 500 IU/l. Diffuse nuclear glycogenization of the hepatocytes may present radiographically as diffuse hepatic steatosis and should be distinguished from nonalcoholic fatty liver disease (NFLD). In this case, a liver biopsy to differentiate these two entities is recommended.^17^^,^^18^

Hypoxic hepatitis is found in approximately 1-2% of adults hospitalized in Intensive Care Units and is associated with approximately 50% of in-hospital mortality.^10^^,^^11^ Management of hypoxic hepatitis involves quick diagnosis and treatment of underlying conditions and comorbidities. Treatment is directed at maintaining normal blood pressure, blood oxygenation, and preventing complications of hypoxic hepatitis, which include hypoglycemia, hyperglycemia, hyperammonemia, hepatopulmonary syndrome, rhabdomyolysis, ischemic pancreatitis, sepsis, and multiple organ failure.^9^^,^^14^^,^^15^ The prognosis of patients with hypoxic hepatitis is poor. The factors that may further worsen the outcome are the presence of septic shock, high serum lactate levels, INR>2, high LDH levels, coexisting acute renal failure, and administration of vasopressors.^9^^,^^10^ Septic shock followed by cardiac failure is the most common cause of death among patients with hypoxic hepatitis.^12^

The frequency of this complication in children, especially with newly diagnosed type 1 diabetes and coexisting ketoacidosis, is unknown. Only individual reports describe this complication in children.^16^^,^^20^^,^^21^

Martin and Tomlinson^20^ described the case of a 13-year-old boy with poorly controlled type 1 diabetes mellitus, symptoms of respiratory distress, impaired consciousness, and hepatomegaly. Laboratory tests showed ALAT 1196 IU/L, ASAT 3969 IU/L, glycated hemoglobin 10.2% (RR: 4-6%), pH 6.92 with final diagnosis of hypoxic hepatitis and glycogenic hepatopathy after the liver biopsy. Szypowska et al.^21^ presented a case of a 10-month-old boy with newly diagnosed type 1 diabetes mellitus, otitis media, with body temperature (39.9^o^C) on admission, pH 6.85, and serum glucose level 261 mg/dl. A spike in serum aminotransferase levels (ALT - 8000 IU/L, AST - 6000 IU/L) was noted on day 3 of hospitalization, later significantly reduced and normalized within a few weeks. Zubkiewicz-Kucharska et al.^16^ described a case of a 3-year-old boy with ketoacidosis and hyperglycemia, pH 7.058, BE -28.3 mmol/l (RR: -2.5 - 2.5 mmol/l), HCO3- 6.3 mmol/l (RR: 21-27mmol/l), hyperglycemia 434 mg/dl, with an increase in serum aminotransferases (AST - 12955 IU/L, ALT - 4328 IU/L) on the fourth day of hospitalization with consequent normalization of the parameters.

All of the children in the cases described above survived.^16^^,^^20^^,^^21^ However, there are isolated reports of death in children hospitalized in Intensive Care Units due to shock or chronic heart failure accompanying hypoxic hepatitis. In these cases, hypoxic hepatitis was not the direct cause of death.^19^

Our patient met all criteria for diagnosing hypoxic hepatitis, which is a form of acute liver injury. The peak of aminotransferases usually occurs 48-72h after the organ insult in hypoxic hepatitis. Thus, it is likely that the increase in AST and ALT at the beginning of the 4th day of hospitalization was associated with the patient’s ketoacidosis and dehydration, detected on admission. The death occurred 24 hours later due to the development of fulminant septic shock and acute liver failure. The infectious origin was most likely the lungs. Although the procalcitonin levels were high, the blood cultures were negative in this setting. The re-evaluating radiologist suggested that the CT scan result was nonspecific for hepatic necrosis, probably due to the early stage of liver injury.

Several risk factors adversely affected the patient's prognosis, such as INR>6, and high levels of lactic acid. In addition, the patient presented to the hospital too late, presenting severe ketoacidosis and significant hyperglycemia. The delay in hospital admission and diagnosis may have been related to the onset of the COVID-19 pandemic. Based on clinical history, the possibility of toxic liver injury was excluded.

## CONCLUSIONS

Hypoxic hepatitis can be a rare but reversible complication of type 1 diabetes.^8^ In severely ill patients, it is an unfavorable prognostic factor and is associated with higher in-hospital mortality.^10^^,^^11^ Hypoxic hepatitis is not included in the official list of complications of diabetic ketoacidosis, and the statistics of its incidence in children are unknown.^18^^,^^19^ Therefore, awareness of this complication, early detection by monitoring aminotransferases, LDH, liver function parameters, and early treatment of underlying diseases is essential for the prognosis of these patients.^6^^,^^18^
